# The Effect of Heparan Sulfate Application on Bone Formation during Distraction Osteogenesis

**DOI:** 10.1371/journal.pone.0056790

**Published:** 2013-02-15

**Authors:** Marie Gdalevitch, Bahar Kasaai, Norine Alam, Bruno Dohin, Dominique Lauzier, Reggie C. Hamdy

**Affiliations:** 1 Division of Orthopedics, Shriners Hospital for Children, Montréal, Quebec, Canada; 2 Department of Pediatric Orthopedic Surgery, Université de St-Etienne, Hôpital Femme-Mère-Enfant, Bron, France; Ghent University, Belgium

## Abstract

Bone morphogenetic proteins (BMPs) are recognized for their ability to induce bone formation *in vivo* and *in vitro*. Their osteogenic and osteoinductive properties are tightly regulated by the secretion of specific BMP antagonists, which have been shown to physically bind and sometimes be blocked by the extracellular proteoglycan heparan sulphate side chains (from hereon referred to as HS). The purpose of this study was to investigate if local application of 5 µg of HS proteoglycan to a bone regenerate site in a mouse model of distraction osteogenesis (DO) can accelerate bone healing and affect the expression of key members of the BMP signaling pathway. DO was performed on the right tibia of 115 adult male wild-type mice. At mid-distraction (day 11), half the group was injected locally with 5 µg of HS, while the other half was injected with saline. The mice were sacrificed at 2 time-points: mid-consolidation (34 days) and full consolidation (51 days). The distracted tibial zone was then collected for analysis by μCT, radiology, biomechanical testing, immunohistochemistry, and histology. While μCT data showed no statistically significant difference in bone formation, the results of biomechanical testing in stiffness and ultimate force were significantly lower in the HS-injected bones at 51 days, compared to controls. Immunohistochemistry results also suggested a decrease in expression of several key members of the BMP signaling pathway at 34 days. Furthermore, wound dehiscence and infection rates were significantly elevated in the HS group compared to the controls, which resulted in a higher rate of euthanasia in the treatment group. Our findings demonstrate that exogenous application of 5 µg of HS in the distracted gap of a murine model had a negative impact on bone and wound healing.

## Introduction

Distraction osteogenesis (DO) is a surgical technique widely used for limb lengthening and bone regeneration for a variety of problems such as trauma, infection or malignancies [1]. Although very successful, one of the major limitations of this technique is the prolonged consolidation phase, during which the patient must wear an internal or external device to maintain the correction until the bone has united [2]. This prolonged process often leads to numerous social, medical and financial complications for the patient and health care system. In order to minimize these complications, a great deal of effort is employed in the bone research field to accelerate the healing process and to stimulate bone formation [3], [4], [5], [6]. Various factors have been investigated including the application of growth factors such as fibroblast growth factor (FGF), transforming growth factor-β (TGF-β), platelet-derived growth factor (PDGF), and bone morphogenetic proteins (BMPs) [7], [8].

Among these, BMPs can potentiate powerful osteogenic effects through their actions on the BMP signaling cascade. Canonical BMP signaling involves the binding of extracellular soluble BMP ligands (e.g. BMP-2, 4, 5, 6 7, 8) to BMP receptors located on the cell membrane (e.g. BMPR-I and –II), which then activate intracellular Smads (e.g. Smad 1, 5, 8) to translocate to the nucleus and activate the transcription of downstream genes [9]. To counterbalance BMP signaling, a number of soluble antagonists such as BMP3, Noggin, Gremlin and Chordin also act on the BMP receptors at the extracellular milieu.

A number of *in vitro* and *in vivo* studies in both animals and humans have shown that recombinant BMPs, specifically BMP2 and BMP7 [4], [10], [11], have osteogenic effects in several conditions associated with poor bone formation. Our group has previously characterized the expression of various members of the BMP pathway (ligands, receptors, downstream target genes and antagonists) in murine and rabbit models of DO, demonstrating their important role in the bone lengthening process [8], [12], [13], [14]. We have also shown that endogenous levels of BMP7 are highly upregulated during DO, peaking during mid-distraction when bone repair and regeneration are most necessitated; and that local administration of exogenous BMP7 increased bone formation within the distracted site of rabbit and mouse models of DO [14], [15].

In humans, large supraphysiological doses of exogenous BMPs have to be administered in order to significantly improve bone growth. These doses can have harmful effects, such as ectopic bone formation and potential for malignancies, notwithstanding the extremely elevated costs related with the use of recombinant BMPs [16], [17], [18], [19]. An alternative strategy to administering exogenous BMPs is to manipulate endogenous BMPs by neutralizing or counteracting the activities of their antagonists, such as Noggin or BMP3. Several methods have been shown to inhibit BMP antagonists, including the use of antibodies, RNA interference or naturally-occurring substances such as the extracellular proteoglycan heparan sulfate, or HS [20], [21].

HS is a membrane-bound proteoglycan [22] that has been previously reported to interact with BMP antagonists as well as BMP ligands to modulate protein activity. HS is an endogenous, commercially available, cost effective and clinically feasible alternative to antibody-mediated or RNAi-mediated gene silencing modulation techniques. Structurally, HS is composed of a core protein and highly sulfated glycosaminoglycan side chains of D-glucuronic acid-*N-*acetyl-D-glucosamine repeats [23]. These negatively charged side chains of HS have been shown to bind a myriad of proteins [24], [25], including soluble BMP ligands (e.g. BMP2, BMP4, BMP7) [26], [27], [28] and BMP antagonists (e.g. Noggin) [29], which can have anti- and pro-osteogenic effects on bone, respectively. Previous *in vitro* studies have proposed two different models for the mechanism of action of how HS can bind BMPs and their secreted antagonists. In the first model, HS is proposed to transport BMPs from cell to cell through restricted diffusion; whereas in the second model, HS was shown to retain BMP antagonists such as Noggin to establish an inverse gradient of BMP activity [20].

*In vivo* studies have also demonstrated that the interaction of HS with BMP antagonists can block the activity of these inhibitors thereby potentiating BMP activity during bone healing [20], [22], [23], [30]. One previous animal study demonstrated that in a rat fracture repair model there was 20% increased bone formation when injected with 5 µg of bone derived HS [31]. Another animal study showed that in a critical size rat cranial defect, 5 µg of embryonically derived HS played an important role in accelerating bone healing by 3 months [32]. Therefore, based on its previously reported therapeutic potential in *in vitro* and *in vivo* studies, we postulated that exogenous application of the naturally-occurring HS, particularly at a dose of 5 µg, may maximize the bioavailability of endogenous BMPs during DO; by inhibiting the action of BMP antagonists, and thus improve bone regeneration in a murine model of DO.

It is important to note, that while some studies have attested to the positive effects of HS on bone regeneration, other reports have showed that HS showed no significant effect on bone [20], [33]. The conflicting data on the role of HS on bone formation and BMP signaling can be explained by a number of reasons, including variations in the sulfation patterns, the microenvironment, and pH/ionic presence of the target tissue. For example, the sulfation pattern of proteoglycans, including HS, can drastically affect their binding affinity to different ligands, resulting in stimulation or inhibition of gene expression [34], [35]. The pH/ionic microenvironment has also been shown to affect the binding affinity of HS [22], [36]. HS tends to have a higher affinity to proteins in the presence of cations (e.g. zinc and copper) [37], whereas its binding affinity decreases in a low cationic presence [38], [39].

In light of this controversy, the purpose of this study was to investigate the effects of exogenous, locally-applied kidney-derived HS in a wild-type mouse model of DO; by examining the effects on (a) bone formation through radiology, microCT and biomechanical testing; and (b) at the molecular level the effect on expression of specific BMP proteins by means of immunohistochemistry.

## Materials and Methods

### 1. Ethics

The McGill University Animal Care Committee approved all experimental procedures (protocol #5162). Throughout surgery, mice were anesthetized using inhaled isoflurane and subcutaneously injected with 0.1 ml of buprenorphine (1 mg/kg-Sigma) for pain management. Animals were monitored once daily immediately after surgery and then 3–4 times per week. During the study, humane endpoints were used in accordance with McGill's standard operating protocol. In case of infection at the surgical site, wound dehiscence, weight loss (>20%) or if the animal became cachectic, had difficulty eating, drinking or moving around freely, or had a Body Condition Score (BCS) less than 2, the animal was euthanized. The mice were euthanized by CO2 asphyxia under general anesthesia at the time of sacrifice. This method is consistent with AVMA (American Veterinary Medical Association) euthanasia guidelines on the use of CO2 as a euthanizing agent.

### 2. Animals

**Figure 1:**
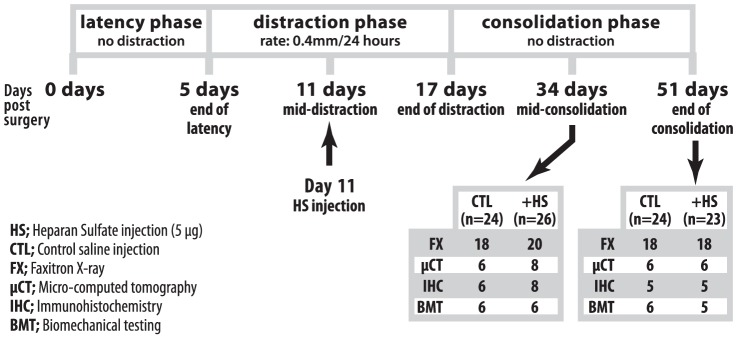
Schematic depiction of distraction osteogenesis procedure, timeline and sample distribution.

Mice were all adult male wild-type C57B16/J mice (Charles River, Montréal, QC), 2–3 months of age with an average weight of 22.0 g (n = 115 for the entire study). Of the 115 mice, 97 mice survived and were processed for analysis. A total of 18 mice were euthanized due to surgical complications: 7 intra-operatively due to fracture and 11 in the post-operative period due to either skin dehiscence, infection or foot necrosis. The samples were sacrificed at two time points (mid-consolidation and full consolidation) and allocated to four groups: faxitron, μCT, immunohistochemistry and biomechanical testing with an objective of having at least 6 samples per group per time point. Due to surgical complications and early euthanizia some groups were left with 5 samples per group. Faxitron was performed on all samples other than the samples allocated for immunohistochemistry (refer to Figure 1 for sample distribution).

### 3. Distraction osteogenesis (DO) procedure

Murine tibial DO was performed using a miniature Ilizarov fixator (Paolo Alto, CA), as previously described by Isefuku *et al.* [5] and our group [12], [40]. Two 0.25-mm pins (Austerlitz, Marlborough, MA) were drilled 90° apart into the proximal and distal metaphysis of the right tibia and secured into position using 2 rings and 8 hexagonal nuts. Three threaded rods were used to connect the two parallel rings. A transverse osteotomy was performed along the middle diaphysis of the right tibia, between the proximal and distal pins, using a no. 11 surgical scalpel (Fisher Scientific, Osaka, Japan). The fibula was then broken using the back end of the scalpel.

Distraction began at a rate of 0.4 mm every 24 hours for 12 days after a 5-day latency period. On post-operative day 11 (mid-distraction), 5 µg of kidney-derived heparan sulfate (HS) (Sigma) diluted into 20 ul of saline, was injected at the distraction site, using a 30-gauge needle. The injections were done at mid-distraction due to the fact that BMP activity is highest during this time and decreases at the beginning of consolidation [12]. The injection technique consisted of using the point of the needle to palpate the tibia from proximal to distal until the needle fell into the distraction gap. The accuracy and reproducibility of this injection technique was previously verified through testing with methylene blue injections in mice that had undergone DO. Control mice were injected with 20 µl of saline alone. The mice were sacrificed at two end points: post-osteotomy day 34 (mid-consolidation) and post-osteotomy day 51 (full consolidation) by CO_2_ asphyxia under general anesthesia. (Refer to Figure 1 for schematic of DO timeline, procedure and sample distribution).

### 4. Tissue Collection

Distracted tissues located between the proximal and distal bone fragments were collected and sent for analysis by Faxitron radiography, microcomputed tomography (μCT), followed by immunohistochemistry and histology. For μCT and radiological imaging, operated tibiae were immersed in 10% buffered formalin and washed with 1× phosphate-buffered saline (PBS) post sacrifice. Soft tissues were not removed from distracted bone specimens to keep the callus intact. Samples were taken to the McGill Centre of Bone & Periodontal Research for μCT and radiological analysis. The same bone samples were then used for immunohistochemistry and histology. Separate samples were sent for biomechanical testing.

### 5. Microcomputed tomography (μCT)

μCT and radiological analysis were performed on distracted tibial samples using the SkyScan 1072 (Aartselaar, Belgium). Distracted tibiae were scanned at 45 KeV/222 µA with 25× magnification (11.25 µm pixel size). Image reconstruction was performed using NRecon (1.4.4, SkyScan). The CT Analyzer (1.8.0.2, SkyScan) was used to measure static histomorphometric parameters of the region of interest, defined as the distracted area located between the proximal and distal bone fragments. The volume of interest for trabecular bone analysis was defined by hand-drawing polygons along the inner surface of cortical bones between the proximal and distal bone fragments. The volume of interest for total bone (including cortical and trabecular bones) analysis is defined by an average estimation of total bone volume in the same gap between the two ends, which is 5.29 cubic mm for all samples. The threshold was determined visually to include all bones to be analyzed. The grayscale indexes ranges from 95–255.

### 6. Faxitron X-ray analysis and Bone-fill score

Faxitron MX-20 (Faxitron X-Ray Corporation, Wheeling, IL) was used to produce radiographs of the distracted samples. Unlabelled radiographs were graded by 3 blinded observers using a 4-point bone fill scoring system, as previously described [3], [41], whereby a score of 0 indicates no bone; a score of 1 accounts for >0% to <50% bone fill; a score of 2 is >50% to <100% bone fill; a score of 3 is a 100% bone fill (see Table 1 for classification of Bone-Fill scores).

**Table 1:** Classification of Bone Fill Scores.

Bone-Fill Scores	Radiographic Evaluation
0	No visible bone-fill (0%)
1	Visible bone-fill, but less than 50%
2	Between 50% and 100% visible bone-fill
3	100% visible bone-fill

### 7. Immunohistochemical protocol and analysis of bone tissue sections

Distracted tibial samples were fixed in 10% buffered formalin overnight, dehydrated in different gradients of acetone, embedded in a mix of methylmethacrylate (MMA) and butylmethacrylate (BMA), and 6 µm sections were made using a Leica RM 2255 microtome (Leica Microsystems, Richmond Hill, ON). Sections were then deplastified, incubated 10 minutes with 3% hydrogen peroxide; then incubated 20 minutes in PBS containing 10% normal horse serum. Distracted tissue sections were probed overnight at 4°C with commercially available polyclonal goat antibodies specific for the target proteins examined, namely BMP-2, -3, -7, Smads 1/5/8, Noggin, Gremlin, Chordin, and BMPR1A (Santa Cruz Biotech.; 1/100 dilution in 1% horse serum). Sections were incubated with biotinylated horse anti-goat secondary antibody (Santa Cruz Biotech. 1/400 dilution in 1% normal horse serum) for 30 minutes, stained using the avidin-biotin complex method 30 minutes; followed by 3,3′-diaminobenzidine (DAB)-peroxidase staining. Sections were then counterstained with hematoxylin and mounted with Permount (Fisher Scientific, Montreal, QC). A negative control was performed for each slide tested by omitting the primary antibody. Pictures of distracted zones were taken under 100× and 400× magnification using a Leica microscope (Leica Microsystems, Richmond Hill, ON) attached to a Q-Imaging camera (Olympus DP70, Japan).

As previously described by our group [12], [14], [42] and others [43], [44], tissue sections of callus were analyzed using a semi-quantitative method for grading positive cell staining and analyzed blindly in triplicates by a single specialist., This semi-quantitative technique scores each immunostained section as a percentage of positively-stained cells compared to total cells, whereby: −represents no staining in the majority of cells; +represents staining in less than 25% of cells; ++represents staining in 25–50% of cells; +++represents staining in 50–75% of cells; ++++represents staining in more than 75% of cells. The analyses were performed for the callus and center of the distracted zone, where *de novo bone* is being consolidated and which consists mainly of chondrocytes and fibroblastic cells (bone cell precursors) [12], [45]. Osteoblastic cells (differentiated bone cells) were either very low in number and/or showed very weak signal intensity in immunostaining during the distraction phase; and were therefore not taken into account for immunohistochemical grading.

### 8. Goldner Trichrome staining of tissue sections

Distracted tibial samples were embedded in a mix of methylmethacrylate (MMA) and butylmethacrylate (BMA), sectioned at 6 µm, then deplastified as described in the above section. Sections were then stained with Goldner-Trichome for comparative histology; and were mounted using Permount (Fisher Scientific, Montréal, QC) for histological analysis. Photomicrographs of distracted zones were taken under 50× magnification to detect for mineralized (green-stained) and non-mineralized (red-stained) regions.

### 9. Biomechanical Testing

Biomechanical testing was performed on samples collected at 34 and 51 days post-surgery and analyzed at the McGill Centre for Bone and Periodontal Research of McGill University (Montreal, Canada). Based on previous studies [46], [47], the three-point bending test was chosen over other methods of biomechanical testing and was conducted using the Mach-1TM Micromechanical Systems device (Bio Syntech Canada, Inc., Laval, QC). The distracted bone was placed on its posterior surface, resting on two supports of a bending apparatus that lie 7 mm apart. A bending load was applied downwards on the mid-shaft of the lengthened tibia at a rate of 50 mm/s until failure. Failure loads were analyzed using the Mach-1TM Motion and Analysis software (version 3.0.2, Bio Syntech Canada). A load-displacement curve was generated using this software to measure biomechanical parameters including stiffness (N/mm), ultimate force (N), ultimate displacement (µm), and work to ultimate failure (N*mm).

### 10. Statistical analysis

All statistical tests for this study were performed using GraphPad Prism version 4.0 (GraphPad Software, La Jolla, CA). Statistical analysis was conducted for histomorphometric parameters, biomechanical testing parameters and bone-fill score using unpaired two tailed t-tests to compare the treated and untreated groups at two separate time points (34 and 51 days). The primary outcome was stiffness (N/mm) at 34 and 51 days and the other parameters were secondary outcomes.

## Results

**Figure 2:**
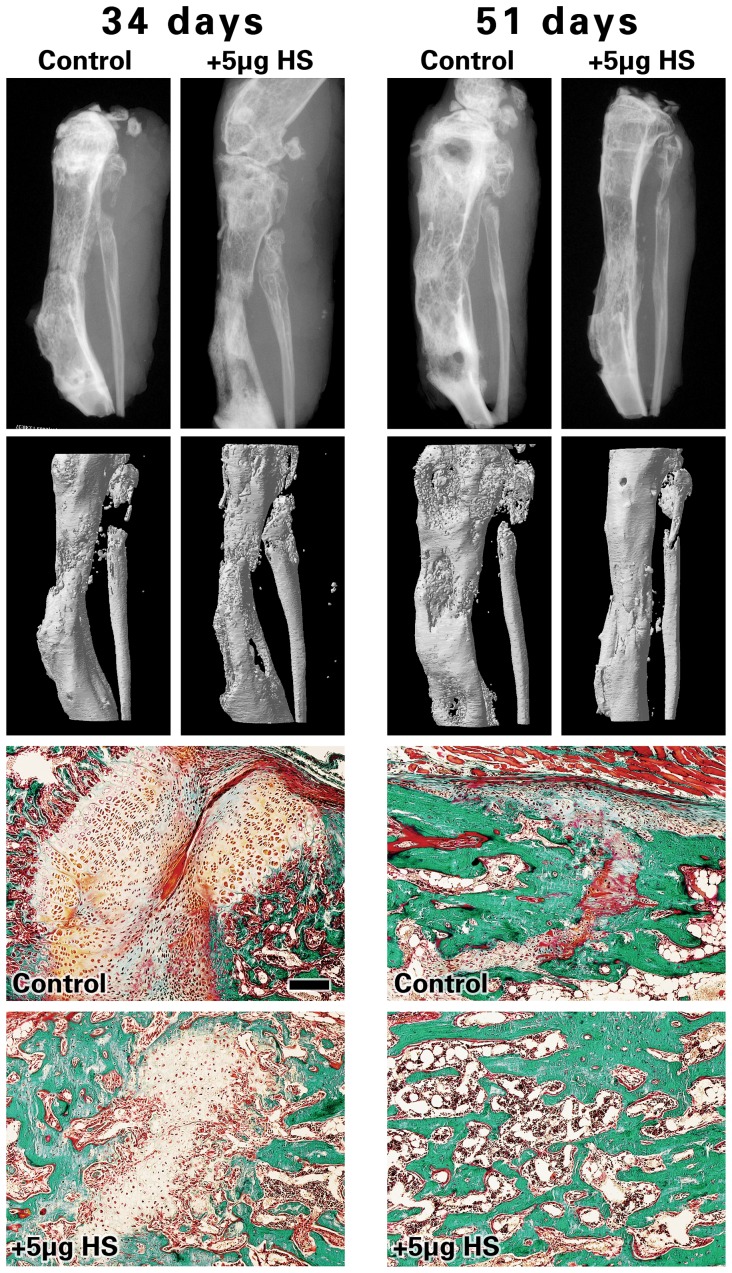
Faxitron, microcomputed tomography (μCT) and histology images. Analysis and radiological images of Faxitron X-ray (top row) and μCT projection (middle-row) of distracted mouse tibiae collected at 34 and 51days post-osteotomy. The third and bottom rows show histology images of distracted tibiae after Goldner-Trichrome staining and reveal regions of the distracted zone that are occupied with soft, connective tissue (red-stained) vs. calcified tissue and/or bone matrix (green-stained). Images were taken at the center of the callus area at 100× magnification (scale bar represents 150 µM).

To determine if HS visibly improves local regenerate healing, the distracted bony tissues were qualitatively assessed via imaging technology. As shown in Figure 2, radiological analysis of Faxitron X-ray (top row) and μCT image projection (middle row), as well as comparative histology via Goldner-Trichrome staining shows qualitative images of *de novo* bone formed in the injected distraction site. There were no gross phenotypic differences between the control and 5 µg HS groups.

### *1. μ*CT static parameters and Bone-Fill scores

**Figure 3:**
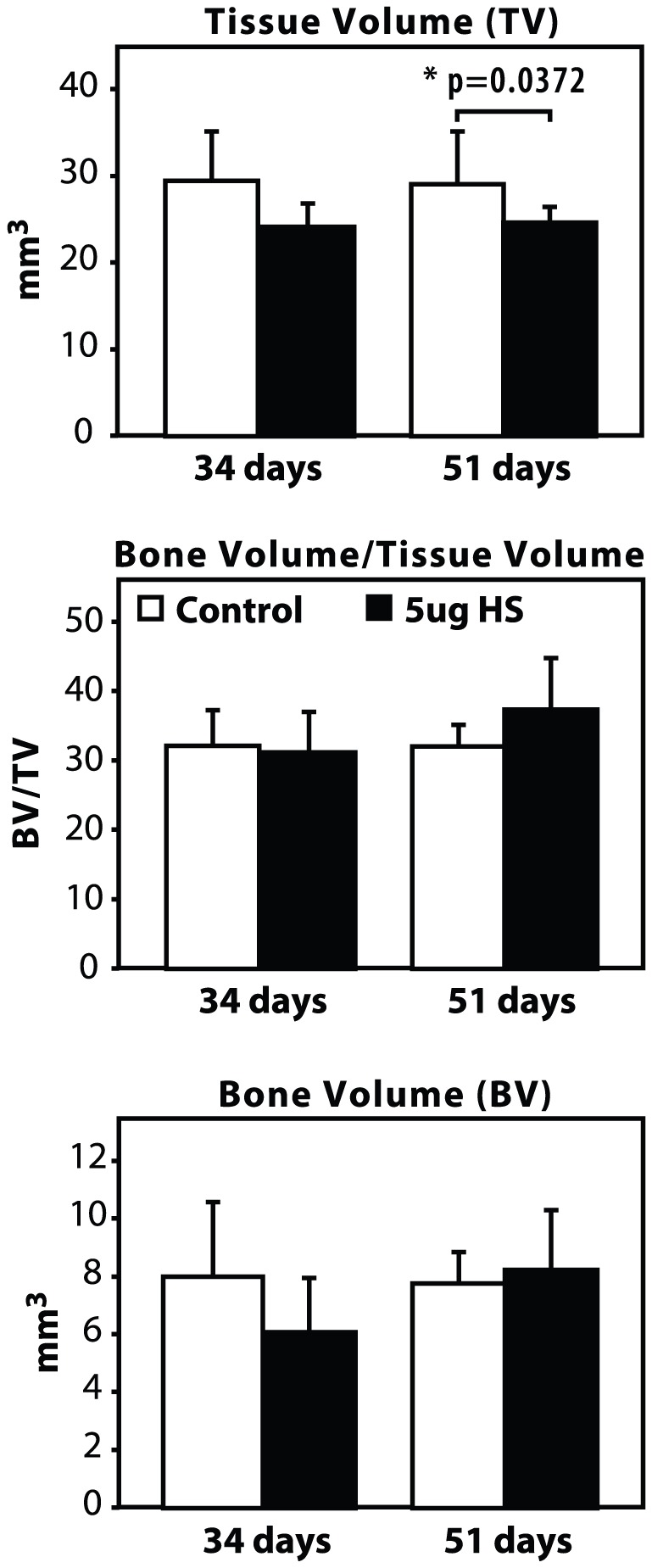
Microcomputed tomography (μCT) results. μCT histomorphometric analysis of distracted bone injected with 5 µg HS or saline (Control), at 34 and 51 days post-osteotomy. For statistical analysis, a two-tailed un-paired t test was performed between the HS-injected group and controls, in which * indicates p<0.05.

**Figure 4:**
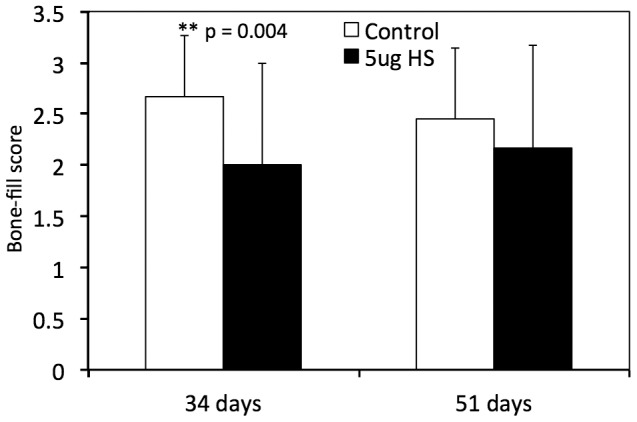
Average bone fill scores. Data is represented as a mean of Bone-fill scores, as blindly graded by radiological assessment.

μCT results showed little to no significant differences in TV (Tissue Volume), BV (Bone Volume), and BV/TV in the HS-injected group compared to controls, at both 34 and 51 days (Figure 3). The only significant finding was that the TV was reduced in the 5 µg HS-injected group (24.58 mm^3^), at 51 days, compared to the controls (29.01 mm^3^, p = 0.0372). It should be noted that while the values for TV, BV and BV/TV were lower at 34 days compared to control, these values were not statistically significant. Bone-fill scores were also reduced in the 5 µg HS-injected bones at 34 days (2.00, compared to 2.67 in control group) and this was statistically significant (p = 0.004). No differences were detected in bone fill scores at 51 days (see Figure 4).

### 2. Biomechanical Parameters of HS-injected bones are reduced in HS group

**Figure 5:**
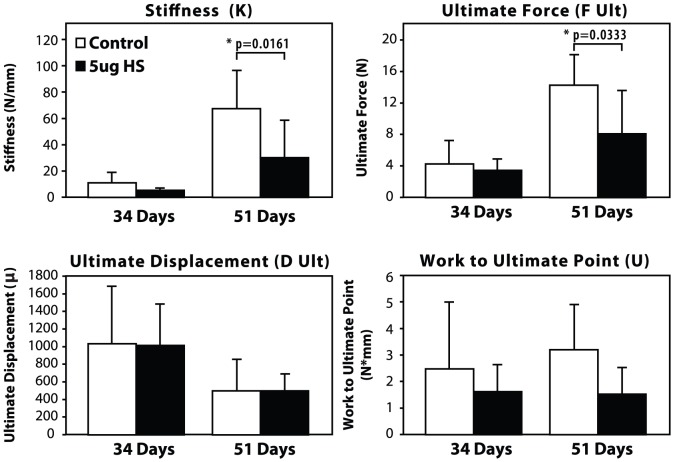
Biomechanical testing results. Biomechanical testing parameters to compare HS-injected bones and saline-injected (control) bones at 34 days and 51 days post-osteotomy. For statistical analysis, a two-tailed un-paired t test was performed between the HS-injected group and controls, in which * indicates p<0.05.

Results of biomechanical testing in Figure 5 show that there were no statistically significant differences in the biomechanical parameters between the groups at 34 days. Stiffness value for the HS-injected group were reduced by about half-fold at 51 days (30.00 N/mm; compared to 67.33 N/mm in control) and was statistically significant (p = 0.016). Similarly, Ultimate Force was reduced in the HS-injected bones at 51 days (8.06 N, compared to 14.24 N in the controls) with statistical significance (p = 0.033). No significant changes were detected in Work to Ultimate Point or Ultimate displacement between groups.

### 3. Post-operative Complications were increased in HS-injected mice

**Figure 6:**
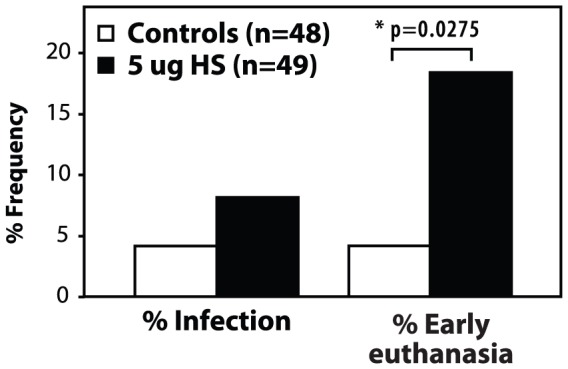
Frequency of post-operative complications. The frequency of infection and early euthanasia was increased in the HS-injected group compared to controls. For statistical analysis, a two-tailed un-paired t test was performed between the HS-injected group and controls, in which * indicates p<0.05.

Importantly, increased complication rates were also observed in the HS group (p = 0.0275), in which 9 mice had to be sacrificed early due to post-operative complications, such as wound dehiscence, foot necrosis and infection (18.4%) compared to 2 mice in the control group (4.2%) as outlined in Figure 6. Infection rates were also increased in HS mice at a frequency of 8.2% compared to 4.2% in the control group, although this was not statistically significant.

### 4. Immunohistochemistry results show down-regulation of members of BMP signaling pathway in HS-injected bones

Of equal interest was whether exogenous HS had any effect on the expression pattern of proteins involved in the osteogenic BMP pathway. Given the large number of proteins directly involved in the BMP pathway, only a select few were chosen to address the objectives of this study for immunohistochemical analysis; namely BMP-2 and 7 for their high osteogenic potential and reported ability to bind HS [26], [27], [28]; Smad 1,5,8 for their actions as intracellular activators of canonical BMP [9]; four well-understood BMP soluble antagonists (Noggin, Gremlin, Chordin, BMP-3) [9], [29]; and BMPR-1A as a well characterized receptor known for its role in bone formation [12], [21]. Indeed, our findings showed that mainly hypertrophic chondrocytes stained positive for these key members of the BMP signaling pathway, namely: BMP-2 and -7 ligands; BMPR1A receptor; Smad 1/5/8; and BMP antagonists BMP3, Gremlin, Noggin and Chordin. While other members of the BMP pathway were also analyzed, the chondrocyte expression levels were either inconclusive (BMPRII, ActRI, Noggin) or insignificant (BMP7, BMPRIa, BMPRIb, ACTRIIa) between HS doses and expression levels (data not shown).

**Figure 7:**
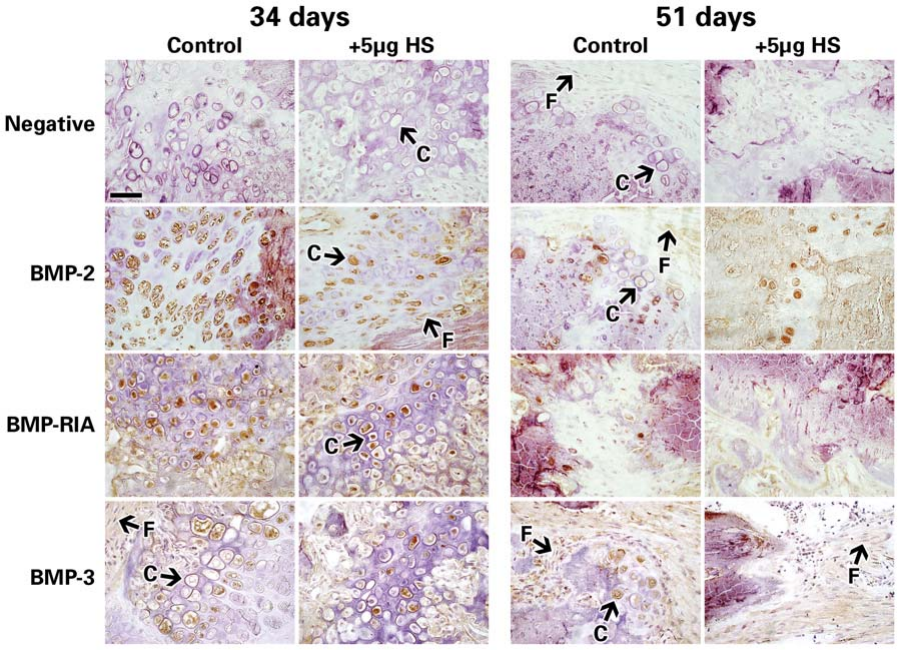
Histochemistry images of distracted mouse tibiae. Mouse tibiae immunostained for members of the BMP signaling pathway (BMP-2, BMPR1a, BMP-3) at 34 and 51 days. Representative images taken at 400× magnification, scale bar represents 50 µM. Chondrocytes and fibroblastic cells are indicated by the white arrows and letters “C” and “F”, respectively.

Figure 7 shows representative immunohistochemistry images of some of the proteins analyzed. The expression levels of these proteins were blindly graded for positive cell staining, and Table 2 shows the summarized results of these immunohistochemistry data. In the callus and distracted zone, chondrocytes and fibroblasts were identified morphologically.

**Table 2:** Summarized results for Immunohistochemistry analysis.

		Control		5 µg HS	
Protein	Endpoint (days)	Chondrocytes	Fibroblasts	Chondrocytes	Fibroblasts
**BMP-2**	34	++	−	+	−
	51	+	+	+	−
**BMP-7**	34	++	−	−	−
	51	+	−	−	−
**Smad 1/5/8**	34	+++	−	+	−
	51	+	−	+	−
**Noggin**	34	++	+	+	−
	51	+	−	+	−
**Gremlin**	34	++	+	+	+
	51	+	−	+	−
**Chordin**	34	++	+	+	+
	51	+	−	+	−
**BMP-3**	34	++	−	+	−
	51	+	−	−	−
**BMPR1A**	34	+++	−	+	−
	51	+	−	−	−

Immunohistochemistry results of dissected tibiae at 34 days and 51 days post-osteotomy.

Specifically, chondrocytes of the distracted tibiae at 34 days post-osteotomy showed decreased expression of Noggin and Chordin (the antagonists) but they also showed a decreased expression of BMP2 and BMP7 as well as all the other tested proteins in the BMP pathway at 34 days, or mid-consolidation (Table 2). At 51 days (full-consolidation), no major change in protein expression levels was detected between the 5 µg HS and control groups (Table 2). Fibroblasts showed little to no positive staining and little to no change between groups and/or endpoints.

## Discussion

To the best of our knowledge we are the first group to study the role of 5 µg of HS proteoglycan specifically in a model of DO. Using our well-established mouse DO model [8], [12], [13], [46], we tested the effects of 5 µg of HS [32], [48] on bone formation at the regenerate site. Our hypothesis that HS binding to BMP antagonists would result in an increase in endogenous BMPs, and subsequently accelerate bone consolidation within the distraction gap, could not be substantiated. In fact, our results suggested the opposite, showing that 5 µg of HS had a negative effect on bone healing and regeneration. We showed that the Bone-fill scores and biomechanical parameters of the regenerate bone formed in the distracted zone were weaker in HS-injected mice compared to controls. We also observed an increase in post-operative complications such as wound dehiscence and skin infection resulting in an increased early euthanasia rate in the HS-injected mice. This implies that bone and wound healing were both negatively affected in the HS treated group.

While μCT analysis showed a decrease in most of the bone morphometric parameters of *de novo* bone in HS-injected mice, these changes were not statistically significant. Conversely, biomechanical testing parameters and bone-fill scores at 51 days post-osteotomy were significantly lower, in the 5 µg HS group compared to the controls. This discrepancy between μCT and biomechanical testing results may be explained by some limitations of the μCT technique. Although μCT measures bone regeneration in a quantitative manner it can be challenging to delineate appropriate thresholds and to accurately define the distraction gap in the small tibia of a mouse. Futhermore, μCT assesses the volume of bone in the gap but cannot determine if it is contiguous or uniforme. The bone volume of the samples between our two groups were similar. However, if the regenerate was not contiguous or uniforme in one group, then this would translate into differences in strength between the groups, thereby explaining the discrepancy between the two assessments. Biomechanical testing describes the functional integrity of the regenerate bone as well as its strength and is a better assessment of the quality of the regenerate. At 51 days (full consolidation), the Stiffness (K) and Ultimate Force (F Ult) scores of the controls were about twice-fold that of the HS group, which were statistically significant (p = 0.0161 and p = 0.0333, respectively).

Our immunohistochemistry results further corroborate the evidence that 5 µg of HS has a negative impact on bone regeneration in our model, since the expression of all 10 of the analyzed proteins involved in the osteogenic BMP signaling pathway (ligands and antagonists alike) were decreased in the 5 µg HS group at 34 days post-osteotomy. Exactly how HS affects BMP signaling is still unclear. One of the known actions of HS is to bind the antagonists of BMPs, such as Chordin and Noggin [29], [49]. Cell-surface HS has been shown to selectively bind and stabilize Noggin and Chordin and to increase their antagonism of BMP signaling [33], [49]. Our immunohistochemistry data showed that 5 µg of exogenous HS resulted in a slight decrease in the endogenous expression of BMP antagonists Noggin, Chordin, Gremlin and BMP3 but also resulted in a slightly decreased expression of endogenous BMP-2 and BMP-7. To account for these findings, we speculate whether HS acts to stabilize the BMP ligand/antagonist interaction rather than modulate their protein expression level and thus prolongs the inhibitory effects of the antagonists on bone formation (and wound healing) during DO. In order to confirm the exact role of HS on the mechanism of BMP signaling activity, HS binding assays would be required but that is outside the scope of the present study.

Another potential explanation is that HS and BMP antagonists may have different binding sites on the BMP ligand [50], [51]. Jackson *et al.* [31] showed that a single dose local application of 5 µg bone-derived HS had an anabolic effect on rat femoral fracture repair after 2 weeks, potentially by increasing the production of local growth factors (ALP, Runx2, FGF-1, IGF-II, TGF-µ1, VEGF). However, similar to our study, HS did not significantly increase BMP-2 or -7 expression. In fact, it has yet to be shown that HS interacts directly with the BMP2 or BMP7/receptor complex.

The delivery method and therapeutic dose of HS that reaches the bone can also influence its effects. A study by Woodruff *et al.* [32], demonstrated that the use of 5 µg of embryonically derived HS, loaded on a scaffold with a more uniform and prolonged distribution of HS, greatly contributed to improve wound healing and bone healing in a rat critical size cranial defect model at 3 months; whereas no difference was demonstrated at 1 month. In our study, we injected HS diluted into saline directly into the regenerate site, a potentially confined space with a surrounding membrane of tissue and as such we may have effectively increased the therapeutic dose of HS over a short period of time. In fact, Jackson *et al.* [31], demonstrated in their dosing study of a rat fracture repair model, that the therapeutic effects of HS can be dose dependant and that a very elevated therapeutic dose can actually have negative effects on bone healing.

Another potential explanation may be related to the pH/ionic microenvironment of the distracted zone, where HS tends to have a lower binding affinity to proteins in acidic milieus [38], [39]. In our model of DO, acidosis in the distracted gap resulting from hypoxia [52] likely caused a decrease in cationic presence in the callus. This acidic microenvironment may have potentiated a decrease in binding affinity of HS to BMP antagonists and resulted in decreased bioavailibilty of endogenous BMPs.

In summary, a large number of factors can influence the binding sites, the specificity and consequently, the structure-function of HS, which in itself makes HS a very difficult therapeutic target. Of great concern, was the increased complication rate observed in the HS group (18.4% vs. 4.2% in the controls), mostly related to wound dehiscence and infection. One possible explanation for this, is that HS affects both bone healing and wound healing, as demonstrated by Woodruff *et al.* [32]. In our study, HS had a negative effect on bone healing and as such may have also had a negative effect on wound healing, secondary to its effects on the surrounding growth factors.

Our findings add to the controversy in the literature as to the effect of HS on bone formation *in vivo*. We are the first group to report negative results on both regenerate strength and wound healing with the application of 5 µg HS in a murine model of DO. In fact, HS may not be a specific enough target for bone healing and also raises certain safety concerns. Future studies could focus on determining the appropriate source, biochemical properties and microenvironment at which HS can actually potentiate an anabolic or catabolic effect in bone. However, the results of our study and a review of the literature demonstrate that due to its unspecific and highly variable binding affinity *in vivo*, HS is a difficult and non-specific therapeutic target for increasing endogenous BMPs. For these reasons, we recommend focusing on other avenues as potential targets for impacting the BMP signaling pathway for bone regeneration.
